# *IKZF1* Variants Predicted Poor Outcomes in Acute Myeloid Leukemia Patients with *CEBPA* bZIP In-Frame Mutations

**DOI:** 10.3390/cancers17152494

**Published:** 2025-07-29

**Authors:** Shunjie Yu, Lijuan Hu, Yazhen Qin, Guorui Ruan, Yazhe Wang, Hao Jiang, Feifei Tang, Ting Zhao, Jinsong Jia, Jing Wang, Qiang Fu, Xiaohui Zhang, Lanping Xu, Yu Wang, Yuqian Sun, Yueyun Lai, Hongxia Shi, Xiaojun Huang, Qian Jiang

**Affiliations:** 1 Peking University People’s Hospital, Peking University Institute of Hematology, National Clinical Research Center for Hematologic Disease, Beijing Key Laboratory of Hematopoietic Stem Cell Transplantation, Peking University, Beijing 100871, China; shunjieyu95@163.com (S.Y.);; 2 Peking-Tsinghua Center for Life Sciences, Academy for Advanced Interdisciplinary Studies, Peking University, Beijing 100871, China; 3 State Key Laboratory of Natural and Biomimetic Drugs, Peking University, Beijing 100871, China; 4 Peking University People’s Hospital, Qingdao 266113, China

**Keywords:** acute myeloid leukemia, *CEBPA* bZIP in-frame, mutation topography, survival prognosis

## Abstract

**Background:** CCAAT/enhancer-binding protein alpha–basic leucine zipper in-frame (*CEBPA*^bZIP-inf^) mutations are associated with favorable outcomes in acute myeloid leukemia (AML). So far, there are limited data on integrating clinical and genomic features impacting the outcomes. **Methods:** Clinical and genomic data from consecutive patients with *CEBPA*^bZIP-inf^ were reviewed. A Cox proportional hazards regression was used to identify the variables associated with event-free survival (EFS), relapse-free survival (RFS) and survival. **Results:** 224 *CEBPA*^bZIP-inf^ patients were included in this study. In the 201 patients, except for the 19 receiving the transplant in the first complete remission with no events (the transplant cohort), multivariate analyses showed that *IKZF1* mutations/deletions were significantly associated with poor EFS (*p* = 0.001) and RFS (*p* < 0.001); *FLT3*-ITD mutations, poor RFS (*p* = 0.048). In addition, increasing WBC count, lower hemoglobin concentration, non-intensive induction, and MRD positivity after first consolidation predicted poor outcomes. On the basis of the number of adverse prognostic covariates for RFS, the 201 patients were classified into low-, intermediate- or high-risk subgroups, and there were significant differences in the 3-year EFS, RFS and survival rates (all *p* < 0.001); however, except for survival in the low-risk group, these metrics were lower than those in the transplant cohort. **Conclusions:** We identified a potential high-risk population with adverse prognostic factors in *CEBPA*^bZIP-inf^ AML patients for which transplantation should be considered.

## Simple Summary

*CEBPA*^bZIP-inf^ mutations are associated with favorable outcomes in acute myeloid leukemia (AML). However, data on the comprehensive integration of clinical data, genetic characteristics, and measurable residual disease in those patients are limited. We found that *IKZF1* mutations/deletions and *FLT3*-ITD mutations predicted poor outcomes in AML patients with *CEBPA*^bZIP-inf^ mutations. And we identified a potential high-risk population with adverse prognostic factors in *CEBPA*^bZIP-inf^ AML patients for which transplantation should be considered.

## 1. Introduction

CCAAT/enhancer-binding protein α (CEBPA) is detected in approximately 10% of patients with acute myeloid leukemia (AML). Recent studies revealed that mutations in the basic leucine zipper region of CEBPA (*CEBPA*^bZIP-inf^) were associated with favorable outcomes regardless of the mono or biallelic status [1,2], which is a favorable risk group per the 2022 European LeukemiaNet (ELN) and International Consensus Classification (ICC) [3,4]. However, certain patients with *CEBPA*^bZIP-inf^ mutations fail to achieve a complete remission (CR), experience relapse, or even die, suggesting clinical and biological heterogeneity.

Several studies have reported that increasing age, higher white blood cell (WBC) count, and non-intensive induction are associated with poor outcomes and that *CSF3R*, *WT1*, *NRAS*, *KIT*, *TET2*, *DNMT3A,* and *FLT3*-ITD mutations predict a high likelihood of relapse; however, most of these mutations were identified by univariate analysis [1,5,6,7]. Even so, the data are limited, and precise risk stratification to identify high-risk subgroups and optimal treatment strategies for patients with *CEBPA*^bZIP-inf^-mutated AML are needed. Therefore, we integrated data from 224 consecutive AML patients with *CEBPA*^bZIP-inf^ mutations to explore the clinical covariates and genetic abnormalities associated with specified outcomes.

## 2. Patients and Methods

### 2.1. Patients

The data of consecutive AML patients with *CEBPA*^bZIP-inf^ mutations from January 2017 to August 2024 who were diagnosed and treated at Peking University People’s Hospital were reviewed. Diagnosis and classification were performed according to the 2022 ELN recommendations for AML [3]. The last follow-up date was January 2025. The study was approved by the Ethics Committee of Peking University People’s Hospital (2021PHB136-001), and written informed consent was obtained from all patients in compliance with the Declaration of Helsinki.

### 2.2. Immune, Cytogenetic, and Molecular Analyses and Next-Generation Sequencing (NGS)

Immunophenotyping was performed using diagnostic bone marrow (BM) aspirate samples, which were analyzed by flow cytometry, as previously described [8]. The karyotype was determined by the G-banding method following the 2013 International System for Human Cytogenetic Nomenclature [9]. Bone marrow aspirates were collected for measurable residual disease (MRD) monitoring by multiparameter flow cytometry (MPFC) after each chemotherapy cycle, and the MRD test sensitivity was 0.01%, as previously reported [8]. NGS was used to detect gene mutations in patients, as previously described [10]. The hematologic tumor panel was shown in Supplemental Table S1. The algorithm independently developed by the Guangzhou Jinyu Company was used to detect variations. A variant allele frequency (VAF) cutoff of 1.0% was used for SNVs and indels.

### 2.3. Treatment

Induction treatment included intensive therapy, such as the “3 + 7” regimen and homoharringtonine combined with the cytarabine and aclarubicin (HAA) regimen, or non-intensive therapy, such as the low-dose cytarabine or anthracycline-based regimen and venetoclax plus azacytidine regimen. The details of the treatment were described in the Supplementary Materials.

### 2.4. Definitions

The response to the first induction therapy was evaluated on days 21–28. For patients whose blood cell counts have not fully recovered, we reevaluated the peripheral blood and BM conditions two weeks later to determine the remission status. CR, CRi, and relapse were defined according to the 2022 ELN recommendations for AML [3]. MRD relapse was defined as the conversion from MRD negativity to MRD positivity (>0.01%) in patients with CR/CRi by MPFC. Events were defined as failure to achieve CR/CRi after 2 cycles of induction therapy, hematologic relapse, MRD relapse, or death from any cause. Event-free survival (EFS) was measured from the time of diagnosis to the date of the first event or the last follow-up. Relapse-free survival (RFS) was calculated as the time from achieving CR/CRi to hematologic relapse, death, or the last follow-up. Survival was calculated from the time of diagnosis of AML to death or the last follow-up.

### 2.5. Statistical Analysis

Baseline characteristics were summarized using descriptive statistical methods. Categorical variables were expressed as frequencies (percentages), whereas continuous measures were summarized using medians with interquartile ranges (IQR). Comparative analyses employed the following appropriate statistical tests: χ^2^ tests or Fisher’s exact tests for categorical variables and Student’s *t*-tests for normally distributed continuous variables. X-tile plots were constructed to determine the optimal cutoff values for continuous covariates for predicting outcomes [11]. Continuous variables without significant prognostic significance are presented as medians. Variables, including clinical and genetic alterations (frequency ≥ 5%), first underwent univariate screening for association with outcomes. Variables demonstrating marginal significance (*p* < 0.20) were subsequently entered into multivariable Cox proportional hazards regression models employing backward elimination. Final models identified independent prognostic variables, with effect estimates expressed as hazard ratios (HRs) accompanied by 95% confidence intervals (CIs). Significant covariates from the multivariate analysis of RFS were used to develop risk stratification. Weighted scores were assigned to these covariates according to the regression coefficient [12]. The risk stratification model underwent internal validation through a bootstrap resampling procedure with 1000 iterations [13,14]. Model discrimination was evaluated by calculating the area under the receiver operating characteristic curve (AU-ROC) [15]. A two-tailed *p*-value < 0.05 was established as the threshold for statistical significance. All statistical analyses were performed using SPSS 26.0 (IBM Corp., Armonk, NY, USA), R statistical environment version 4.0.2 (R Foundation for Statistical Computing, Vienna, Austria), and GraphPad Prism 8 (GraphPad Software, San Diego, CA, USA), with the latter also employed for data visualization.

## 3. Results

### 3.1. Patient Characteristics

The data of 224 consecutive *CEBPA*^bZIP-inf^ mutated AML patients were reviewed. A total of 124 (55%) patients were male. Median age was 42 years (IQR, 32–54 years). A total of 58 (26%) patients had cytogenetic abnormalities, including 15 with adverse cytogenetic risk. A total of 194 patients received intensive induction chemotherapy, and 30 received non-intensive induction chemotherapy (Table 1). Among the 14 patients with *FLT3*-ITD mutations, 9 received chemotherapy combined with sorafenib from induction therapy, and 1 received sorafenib after relapse.

### 3.2. Mutation Topography

**Figure 1:**
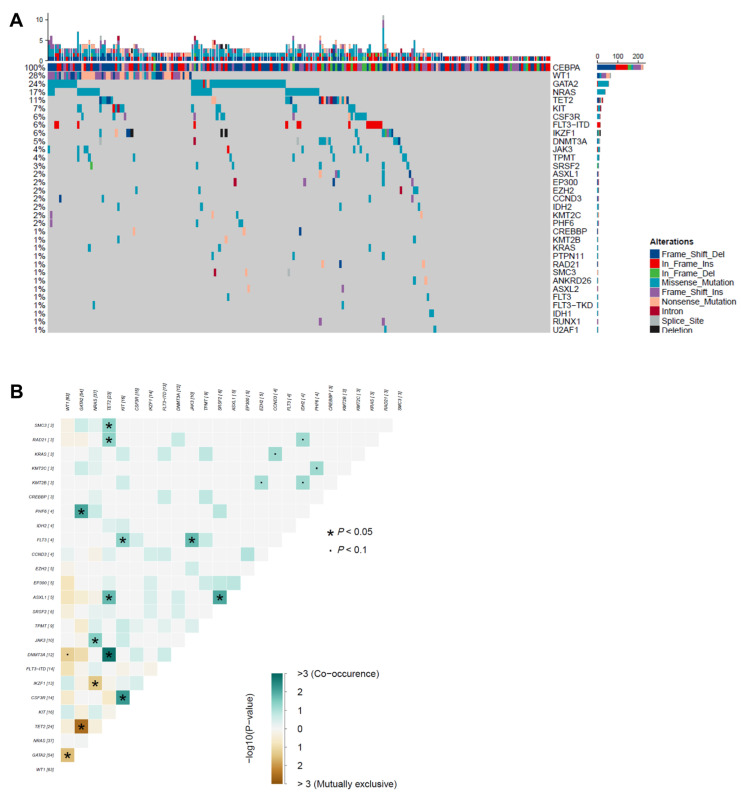
Mutation topography in AML with *CEBPA*^bZIP-inf^ mutation. (**A**) Co-mutations. Each column indicates the data of one sample; each row indicates a gene. The top bar indicates the mutation frequency (mutation/Mb DNA), and the right bar indicates the frequency of different mutated genes. Sorted by frequency of mutations in the entire *CEBPA*^bZIP-inf^ AML patients. (**B**) Pairwise associations between gene mutations. Gene interaction patterns were assessed through pairwise co-occurrence analysis. Fisher’s exact tests quantified association strength between gene pairs, with odds ratios (OR) and corresponding *p*-values computed under the null hypothesis of independent distribution. Associations reaching significance threshold (* *p* < 0.05, ∙ *p* < 0.01) underwent visual encoding using a two-dimensional matrix. Forest green hues signified synergistic co-occurrence (OR > 1), whereas amber tones indicated mutual exclusivity (OR < 1).

By targeted sequencing, additional mutations were identified in 178 of 224 (79%) patients with *CEBPA*^bZIP-inf^ mutations. The most frequently mutated genes were *WT1* (n = 63, 28%), followed by *GATA2* (n = 54, 24%), *NRAS* (n = 37, 17%), *TET2* (n = 24, 11%), *KIT* (n = 16, 7%), *FLT3*-ITD (n = 14, 6%), *CSF3R* (n = 14, 6%), *IKZF1* mutations (n = 10) and deletions (n = 3, 6%), and *DNMT3A* (n = 12, 5%) (Figure 1A). Except for that in *CEBPA*, the median mutation number per patient was two (IQR, 1–3; range, 0–11). When the co-occurrence and mutual exclusivity patterns of mutations with frequencies > 5% were explored, we found co-mutations in *CSF3R* and *KIT* and in *DNMT3A* and *TET2*. *GATA2* mutations and *WT1* or *TET2* mutations were mutually exclusive, as were those in *IKZF1* and *NRAS* (Figure 1B).

### 3.3. Outcomes

Among the 224 AML patients, 204 (91%) achieved CR/CRi after the first induction therapy, and 221 (99%) ultimately achieved CR/CRi; 69 (31%) showed MRD positivity after induction therapy, and 27 (12%) showed MRD positivity after the first consolidation therapy. With a median follow-up of 24 (IQR, 13–42) months for all patients and 25 (IQR, 12–43) months for survivors, 29 (13%) and 80 (36%) experienced MRD and hematologic relapse, respectively; 74 (33%) underwent a transplant, including 19 in CR1 with MRD negativity based on their preference (the transplant cohort), 18 in CR1 with MRD positivity, and 37 in CR2. A total of 30 (13%) patients died of no response (n = 1), hematologic relapse (n = 25), severe infection (n = 2), or transplantation-related mortality (n = 2). The baseline characteristics of the transplant cohort are presented in Table 1. There were some differences in the patients’ characteristics at diagnosis between the transplant and nontransplant cohorts, including younger age (*p* < 0.001), higher WBC counts (*p* = 0.015), higher BM blast proportions (*p* = 0.012), and more *KIT* (*p* = 0.003) and *FLT3*-ITD (*p* = 0.033) mutations were observed in the transplant cohort compared to the nontransplant cohort. Notably, no events occurred in the transplant cohort during the follow-up period. The 3-year probabilities of EFS (100% vs. 38% [95% CI [31, 47%], *p* < 0.001), RFS (100% vs. 48% [41, 57%], *p* < 0.001), and survival (100% vs. 80% [72, 87%], *p* = 0.024) were higher in the transplant cohort than in the nontransplant cohort.

### 3.4. Impact of Co-Mutations and Cytogenetic Abnormalities on Outcomes

**Figure 2:**
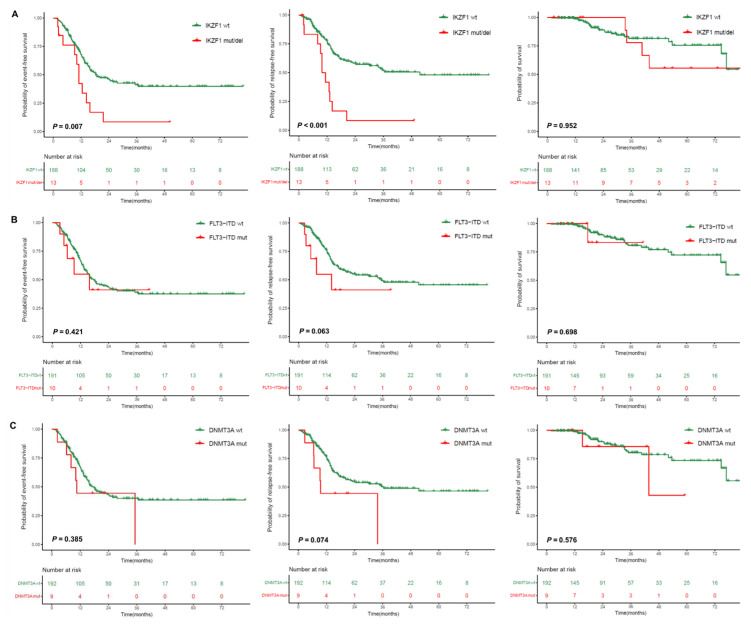
Impact of co-mutations on outcomes. (**A**) *IKZF1* mutations/deletions; (**B**) *FLT3*-ITD mutations; (**C**) *DNMT3A* mutations.

Because no events occurred in the transplant cohort during the follow-up period, we explored the effects of clinical covariates and genetic abnormalities (frequencies ≥ 5%) on outcomes in the nontransplant cohort for which MRD data were available. Univariate analyses revealed that *IKZF1* mutations/deletions were associated with poor EFS (*p* = 0.007) and RFS (*p* < 0.001); *FLT3*-ITD mutations and *DNMT3A* mutations had a tendency toward adverse RFS (*p* = 0.063 and *p* = 0.074) (Figure 2). Other common co-mutations had no prognostic significance. Notably, myelodysplasia-related gene mutations and cytogenetic abnormalities had no impact on outcomes (Supplemental Table S2).

The frequency of *IKZF1* mutations (n = 10) or deletions (n = 3) was 6%, with a median VAF of 24% (range: 2–45%) observed in the mutated patients. Missense mutations were the predominant alterations (n = 4), followed by frameshift deletions (n = 3), in-frame deletions (n = 1), nonsense mutations (n = 1), and frameshift insertions (n = 1) (Supplemental Figure S1). In addition, three patients had *IKZF1* exon 4–8 deletion.

### 3.5. Identifying Covariates Associated with Outcomes

In the 201 patients (the nontransplant cohort), clinical variables that may be related to outcomes, including baseline characteristics (age, sex, complete blood count, BM blasts, cytogenetic abnormalities, and genomic abnormalities [frequencies ≥ 5%]), induction chemotherapy, and MRD after induction and first consolidation, were explored. The univariate analysis results are displayed in Supplemental Table S2. In the multivariate analyses, *IKZF1* mutations/deletions were significantly associated with poor EFS (HR  =  3.0 [95% CI 1.6, 5.8], *p* = 0.001) and RFS (HR = 3.8 [1.9, 7.3]); *FLT3*-ITD mutations, poor RFS (HR = 2.5 [1.0, 6.5], *p* = 0.048). In addition, increasing WBC count was significantly associated with poor EFS (HR = 1.8 [1.2, 2.7], *p* = 0.003), RFS (HR = 2.0 [1.3, 3.0], *p* = 0.002), and survival (HR = 1.7 [0.8, 3.5], *p* = 0.038); non-intensive induction, poor EFS (HR = 3.2 [1.8, 5.6], *p* < 0.001) and RFS (HR = 3.9 [2.2, 6.9], *p* < 0.001); MRD positivity after first consolidation, worse RFS (HR = 2.4 [1.3, 4.2], *p* = 0.005) and survival (HR = 4.9 [2.2, 11.2], *p* < 0.001); and increasing HGB concentration, favorable RFS (HR = 0.1 [0.01, 0.3], *p* = 0.001) (Table 2). Using X-tile, we determined that the optimal cutoff values for the WBC count were 32, 69, and 45 × 10^9^/L for EFS, RFS, and survival, respectively, and the HGB concentration was 72 g/L for RFS. In multivariate analyses, these categorical covariates impacting outcomes were still significantly associated with outcomes (Supplemental Table S3).

Next, we analyzed patients according to the induction regimen. In 172 patients receiving intensive induction therapy, the results were similar to those of all patients except for an increased WBC count, which had no impact on survival (Supplemental Table S4). Analysis was not performed for those receiving non-intensive induction therapy because of the small number of patients.

### 3.6. Risk Stratification

**Figure 3:**
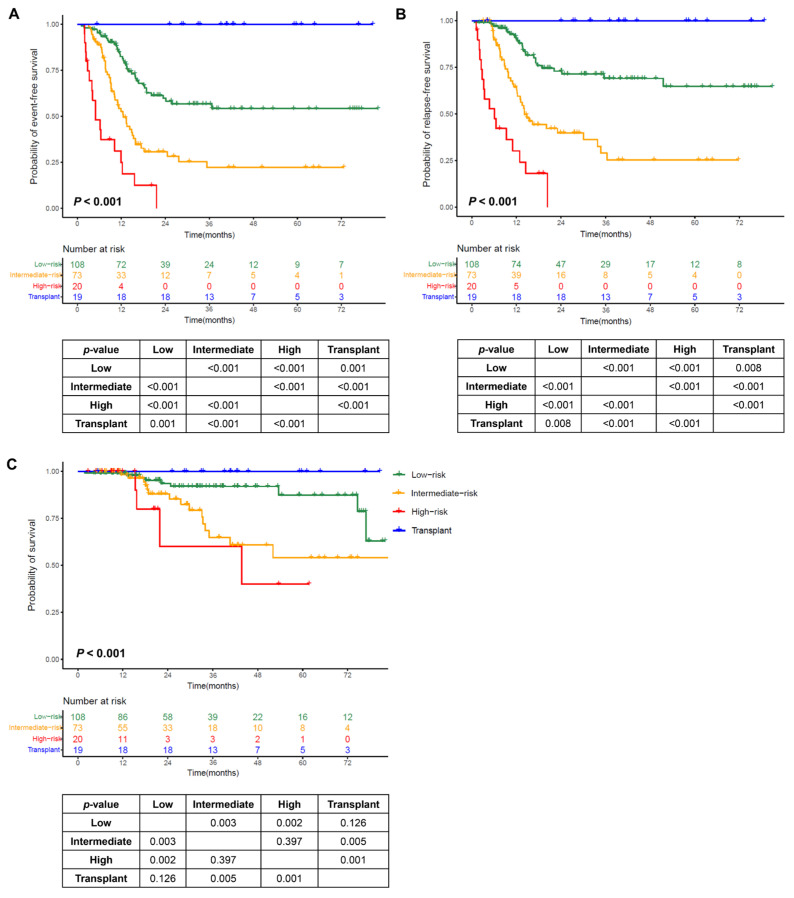
Outcomes by the number of adverse prognostic covariates. (**A**) Event-free survival (EFS); (**B**) relapse-free survival (RFS); (**C**) survival.

Patients in our cohort had a higher relapse rate, so we selected adverse variables affecting RFS to develop the risk stratification. In the 201 patients (the nontransplant cohort), we used the number of adverse prognostic covariates (including WBC count ≥ 69 × 10^9^/L, HGB ≤ 72 g/L, non-intensive induction, MRD positivity after the first consolidation, *FLT3*-ITD mutations, and *IKZF1* mutations/deletions; points were assigned to these covariates according to the regression coefficient, each scored as 1 point) for RFS to divide the patients into low- (score 0; n = 108; 54%), intermediate- (score 1; n = 73; 36%), and high-risk (score ≥ 2; n = 20; 10%) subgroups. Combining the transplant cohort, there were significant differences in EFS, RFS, and survival among the four subgroups (all *p* values for trends <0.001). The transplant cohort had the best outcomes. Differences in EFS and RFS were detected between the subgroups. There was no difference in survival between the intermediate- and high-risk subgroups, and there was no difference between the low-risk subgroup and the transplant cohort, which was significantly greater than that in the intermediate- and high-risk subgroups (Figure 3).

To evaluate the risk stratification performance for predicting outcomes, we plotted ROC curves for the probabilities of RFS at 1, 2, and 3 years (Supplemental Figure S2). The risk stratification showed good sensitivity and specificity with an AUROC value of 0.74 (0.65, 0.81) for 1-year RFS, 0.73 (0.64, 0.80) for 2-year RFS, and 0.74 (0.64, 0.82) for 3-year RFS.

**Figure 4:**
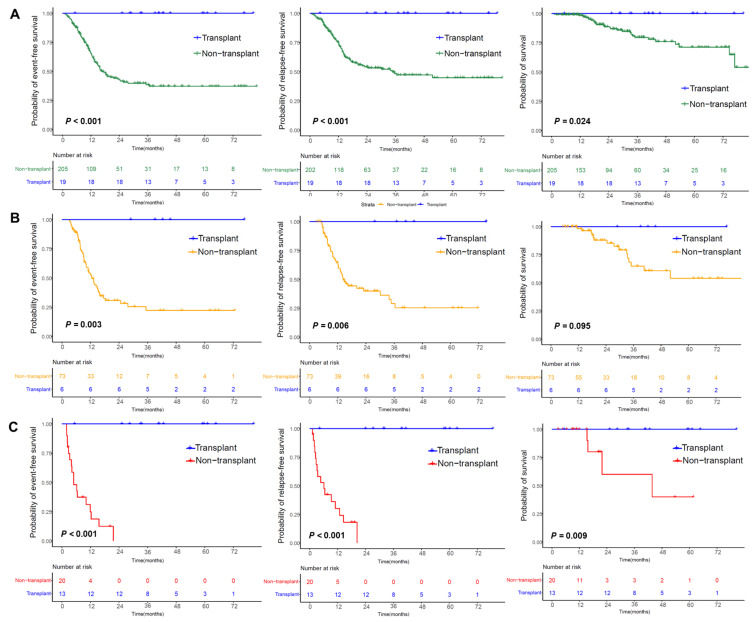
Outcomes in patients with *CEBPA*^bZIP-inf^ mutations. (**A**) Outcomes in the nontransplant cohort and transplant cohort. (**B**) Outcomes in the intermediate-risk subgroup of the nontransplant cohort and transplant cohort. (**C**) Outcomes in the high-risk subgroup of the nontransplant cohort and transplant cohort.

In the transplant cohort, 19 patients were divided into intermediate-risk (n = 6) and high-risk (n = 13) subgroups on the basis of risk classification. The 3-year probabilities of EFS (*p* values < 0.001–0.003), RFS (*p* values < 0.001–0.006), and survival (*p* values = 0.009) in the transplant cohort were higher than those in the nontransplant cohort, except for a tendency toward poor survival (*p* = 0.095) in the intermediate-risk subgroup. (Figure 4A–C).

## 4. Discussion

In this study, we identified *IKZF1* mutations/deletions (6%) as an independent risk factor for worse EFS and RFS in AML patients with *CEBPA*^bZIP-inf^ mutations, which has rarely been reported. *IKZF1* alterations are relatively common (30–50%) in adult acute lymphoblastic leukemia (ALL) patients [16,17], and the most common pattern of alteration is heterozygous deletion of either the whole gene or specific exons with subsequent loss of function [18]. In numerous studies, *IKZF1* mutation was reported to be an independent marker of adverse risk in ALL patients [18,19,20]. However, studies on *IKZF1* alterations in AML patients are limited, with frequencies of 2–5%, which are much lower than those reported in ALL patients, although these frequencies are also associated with poor outcomes [21,22,23].

Consistent with previous studies, *FLT3*-ITD mutations predicted a high likelihood of relapse and worse survival through univariate analysis in AML patients (did not receive *FLT3* inhibitors) with *CEBPA*^bZIP-inf^ mutations or with biallelic *CEBPA* mutations [6,24]. In our study, multivariate analysis revealed that the *FLT3*-ITD mutation was significantly associated with poor RFS. AML with *FLT3*-ITD (without adverse-risk genetic lesions) is classified as intermediate risk [25], but there is limited evidence to support the reclassification of AML with *CEBPA*^bZIP-inf^- *FLT3*-ITD mutations. Although *FLT3* inhibitors significantly improve outcomes in AML patients with *FLT3* mutations [26], whether they can improve the prognosis of patients with AML *CEBPA*^bZIP-inf^ with *FLT3* mutations needs further investigation.

Consistent with previous studies [6,7,27], higher WBC count and MRD positivity after the first consolidation were associated with poor outcomes in our study, and patients may have benefited from intensive induction therapy. Moreover, myelodysplasia-related gene mutations or cytogenetic abnormalities had no prognostic significance in AML patients with *CEBPA*^bZIP-inf^ mutations.

On the basis of the adverse covariates of RFS identified in this study, all 19 patients in the transplant cohort classified in the intermediate- or high-risk subgroup had no events during the follow-up period, whereas those receiving consistent chemotherapy had a high likelihood of relapse and death, suggesting that transplantation might improve the outcomes in AML patients with *CEBPA*^bZIP-inf^ mutations. Although transplantation is not recommended as an essential treatment strategy for patients with *CEBPA*^bZIP-inf^-mutated AML, our findings suggest that intermediate- and high-risk AML patients with *CEBPA*^bZIP-inf^ mutations should consider transplantation.

Our study has several limitations. First, it was a retrospective study. Second, the chemotherapy regimens used were not uniform, and not all patients with *FLT3*-ITD mutations were treated with *FLT3* inhibitors. Third, the number of patients in our study, especially the transplant cohort, was relatively small, and there might be selection bias, which might influence the results and a prospective study is needed to validate the conclusions.

## 5. Conclusions

*IKZF1* mutations/deletions and *FLT3*-ITD mutations predicted poor outcomes in AML patients with *CEBPA*^bZIP-inf^ mutations in addition to a high WBC count, lower hemoglobin concentration, non-intensive induction therapy, and MRD positivity after the first consolidation. We identified a potential high-risk population for transplantation that should be considered. Our findings need to be verified in future studies.

## Figures and Tables

**Table 1:** Patients’ characteristics.

	Total (n = 224)	Nontransplant (n = 205)	Transplant (n = 19)	*p* Value
Male sex, n (%)	124 (55%)	111 (54%)	13 (68%)	0.231
Age, years, median [Q1, Q3]	42 (32, 54)	44 (33, 55)	30 (25, 38)	<0.001
AML history, n (%)				0.989
De novo AML	218 (97%)	199 (97%)	19 (100%)	
Secondary AML	6 (3%)	6 (3%)	0 (0%)	
WBC, ×10^9^/L, median [Q1, Q3]	14.3 (7.6, 45.4)	13.4 (7.3, 40.9)	31.8 (17.9, 77.2)	0.015
HGB, g/L, median [Q1, Q3]	100.5 (84.0, 114.8)	102.0 (84.0, 116.0)	97.0 (82.0, 113.0)	0.530
PLT, ×10^9^/L, median [Q1, Q3]	31.0 (17.0, 56.0)	30.0 (16.0, 55.5)	39 (17.0, 71.0)	0.253
BM blast%, median [Q1, Q3]	59.3 (46.0, 73.0)	58.0 (45.3, 71.0)	71.5 (58.0, 79.0)	0.012
Cytogenetic risk				0.353
Normal	165 (74%)	153 (75%)	12 (63%)	
Intermediate	44 (20%)	38 (19%)	6 (32%)	
Adverse	15 (7%)	14 (7%)	1 (5%)	
*WT1* mutations	63 (28%)	56 (27%)	7 (37%)	0.377
*GATA2* mutations	54 (24%)	49 (24%)	5 (26%)	1.000
*NRAS* mutations	37 (17%)	31 (15%)	6 (32%)	0.127
*TET2* mutations	24 (11%)	23 (11%)	1 (5%)	0.678
*KIT* mutations	16 (7%)	11 (5%)	5 (26%)	0.003
*FLT3*-ITD mutations	14 (6%)	10 (5%)	4 (21%)	0.033
*CSF3R* mutations	14 (6%)	11 (5%)	3 (16%)	0.193
*IKZF1* mutations/deletions	13 (6%)	13 (6%)	0 (0%)	0.536
*DNMT3A* mutations	12 (5%)	11 (5%)	1 (5%)	1.000
Myelodysplasia-related gene mutations	16 (7%)	14 (7%)	2 (11%)	0.894
Induction therapy, n (%)				0.150
Intensive	194 (87%)	175 (85%)	19 (100%)	
Non-intensive	30 (14%)	30 (15%)	0 (0%)	
Follow-up time of survivor, months, median [Q1, Q3]	25 (12, 43)	22 (11, 39)	42 (33, 61)	<0.001

BM, bone marrow; HGB, hemoglobin; PLT, platelets; WBC, white blood cell.

**Table 2:** Multivariate analyses of outcomes.

	EFS		RFS		Survival	
	HR (95%CI)	*p* Value	HR (95%CI)	*p* Value	HR (95%CI)	*p* Value
WBC ^a^	1.8 (1.2–2.7)	0.003	2.0 (1.3–3.0)	0.002	1.7 (0.8–3.5)	0.038
HGB ^a^			0.1 (0.01–0.3)	0.001		
Non-intensive induction	3.2 (1.8–5.6)	<0.001	3.9 (2.2–6.9)	<0.001		
MRD positivity after first consolidation			2.4 (1.3–4.2)	0.005	4.9 (2.2–11.2)	<0.001
*FLT3*-ITD mutations			2.5 (1.0–6.5)	0.048		
*IKZF1* mutations/deletions	3.0 (1.6–5.8)	0.001	3.8 (1.9–7.3)	<0.001		

^a^ log10 transformed. CI, confidence interval; HR, hazard ratio; WBC, white blood cell; HGB, Hemoglobin; MRD, measurable residual disease.

## Data Availability

Data are available on reasonable request from the corresponding authors and consistent with the laws of China.

## Supplementary Materials

The following supporting information can be downloaded at https://www.mdpi.com/article/10.3390/cancers17152494/s1: Supplemental Table S1: List of 290 genes targeted by next generation sequencing panel; Supplemental Table S2: Univariable analyses of outcomes; Supplemental Table S3: Multivariate analyses of outcomes; Supplemental Table S4: Multivariate analyses of outcomes in patients receiving intensive induction therapy; Supplemental Figure S1: Lollipop plot illustrating *IKZF1* mutations; Supplemental Figure S2: ROC curves of the risk stratification for the 1-, 2- and 3-year probabilities of RFS.
