# Supplementary material for: IKZF1 Variants Predicted Poor Outcomes in Acute Myeloid Leukemia Patients with CEBPA bZIP In-Frame Mutations
Source: Cancers (Basel). 2025 Jul 29;17(15):2494. doi: 10.3390/cancers17152494 (PMC12346723; doi:10.3390/cancers17152494)
Supplement: Supplementary file 1 [file cancers-17-02494-s001.zip › cancers-3720491-supplementary.pdf]

Supplemental Materials

Treatment

The intensive induction therapy regimens were "3+7" regimens and HAA regimen (homoharringtonine 2 mg/m^2/day, d1-7; aclarubicin 20 mg/day, d1-7; cytarabine 100 mg/m^2/day, d1-7). The nonintensive induction therapy regimens included CAG regimen (aclacinomycin 20 mg/day, d1-4; cytarabine 10 mg/m^2 every 12 h for 14 days; granulocyte colony stimulating factor [G-CSF], 5 μg/kg/day for 14 days according to the WBC counts) and venetoclax plus azacytidine regimen (oral venetoclax 100 mg d1, 200 mg d2, 400 mg d3-28; azacitidine 75 mg/m^2 was administered IV or subcutaneously on d1-7). Patients who achieved CR with complete hematologic recovery or incomplete hematologic recovery (CR/CRi) received consolidation therapy consisting of high-dose cytarabine-based regimens for 3 or 4 cycles. If the patients did not achieve CR/CRi after 1 or 2 courses of induction or relapse, intermediate- or high-dose cytarabine-based regimens followed by transplant were considered. For unfit patients, less intensive therapies were continued as maintenance therapy.

Supplemental Table S1. List of 290 genes targeted by next generation sequencing panel

ABCB1	*CALR*	*DKC1*	*GNAS*	*LMO2*	*PHF6*	*SETBP1*	*TPMT*
ABL1	*CARD11*	*DNAH10*	*GNB1*	*LTB*	*PIGA*	*SETD1B*	*TRAF2*
AKT3	*CBL*	*DNM2*	*GSKIP*	*LYN*	*PIK3CA*	*SETD2*	*TRAF3*
ALK	*CBLB*	*DNMT3A*	*HAX1*	*MAP2K1*	*PIK3CD*	*SETDB1*	*TRAF5*
ANKRD26	*CBLC*	*DOT1L*	*HFE*	*MAX*	*PIK3R1*	*SF1*	*TYK2*
APC	*CCND1*	*DTX1*	*HIST1H1B*	*MCL1*	*PIM1*	*SF3A1*	*U2AF1*
ARID1A	*CCND3*	*DUSP2*	*HIST1H1C*	*MDM2*	*PIM2*	*SF3B1*	*U2AF2*
ARID1B	*CCR4*	*EBF1*	*HIST1H1D*	*MED12*	*PLCG1*	*SGK1*	*UBR5*
ARID2	*CD22*	*EED*	*HIST1H1E*	*MEF2B*	*PLCG2*	*SH2B3*	*VHL*
ARID5B	*CD28*	*EGFR*	*HRAS*	*MFHAS1*	*PML*	*SH2D1A*	*WAS*
ASXL1	*CD58*	*EGLN1*	*HUWE1*	*MGA*	*PMS2*	*SLC29A1*	*WHSC1*

ASXL2	CD70	EGR1	ID3	MLH1	POT1	SMARCA4	WT1
ATG2B	CD79A	EGR2	IDH1	MPL	POU2AF1	SMARCB1	XPO1
ATM	CD79B	ELANE	IDH2	MSH2	PPM1D	SMC1A	ZAP70
ATP6V1B2	CDC25C	EP300	IGLL5	MSH6	PRDM1	SMC3	ZFHX4
ATRX	CDK6	EPHA7	IKBKB	MTOR	PRF1	SOCS1	ZMYM3
AXIN1	CDKN1A	EPOR	IKZF1	MYC	PRKCB	SP140	ZRSR2
B2M	CDKN1B	ERBB3	IKZF2	MYD88	PRKD2	SPEN	
BAX	CDKN2A	ERG	IKZF3	MYOM2	PRKDC	SRP72	
BCL10	CDKN2B	ETNK1	IL7R	NBN	PRPF8	SRSF2	
BCL11B	CEBPA	ETV6	IRF4	NF1	PTCH1	STAG2	
BCL2	CEBPE	EZH2	IRF8	NFKBIA	PTEN	STAT3	
BCL6	CHD2	FAM46C	ITK	NFKBIE	PTPN11	STAT5A	
BCL7A	CHD8	FAS	ITPKB	NOTCH1	PTPN2	STAT5B	
BCOR	CHEK2	FAT1	JAK1	NOTCH2	PTPN6	STAT6	
BCORL1	CIC	FAT3	JAK2	NOTCH3	PTPRD	SUZ12	
BIRC3	CIITA	FAT4	JAK3	NOTCH4	PTPRT	SYK	
BLM	CREBBP	FBXO11	JUNB	NPM1	RAD21	TAL1	
BLNK	CRLF2	FBXW7	KAT6A	NRAS	RAD50	TBL1XR1	
BPGM	CSF1R	FGFR3	KDM6A	NT5C2	RARA	TCF3	
BRAF	CSF3R	FLT3	KIT	NTRK1	RB1	TERC	
BRCA1	CTCF	FOXO1	KLF2	NTRK2	RELN	TERT	
BRCA2	CUX1	G6PC3	KLHL6	NTRK3	RHOA	TET1	
BRD4	CXCR4	GATA1	KMT2A	NUDT15	RPL10	TET2	
BRIP1	CYLD	GATA2	KMT2B	P2RY8	RPS15	TINF2	
BTG1	DDX3X	GATA3	KMT2C	PALB2	RRAGC	TMEM30A	
BTG2	DDX41	GFI1	KMT2D	PAX5	RTEL1	TNFAIP3	
BTK	DHFR	GNA13	KRAS	PDGFRA	RUNX1	TNFRSF14	
CACNA1H	DIS3	GNAI2	KRT20	PDGFRB	SBDS	TP53	

The hematologic tumor panel containing 175 or 290 genes was used from January 2017 to November 2021 or after December 2021. The red marker gene is the panel of 175 genes.

Supplemental Table S2. Univariable analyses of outcomes

	EFS		RFS		Survival	
	HR (95%CI)	*P* value	HR (95%CI)	*P* value	HR (95%CI)	*P* value
Male (ref. female)	1.1 (0.7-1.6)	0.768	1.2 (0.8-1.8)	0.473	1.3 (0.6-2.7)	0.517
Age[a]	1.1 (1.0-1.3)	0.080	1.2 (1.0-1.4)	0.014	1.2 (0.9-1.6)	0.171
WBC[b]	1.0 (1.0-1.1)	0.001	1.1 (1.0-1.1)	<0.001	1.0 (1.0-1.1)	0.049
HGB[c]	0.9 (0.8-1.0)	0.035	0.9 (0.8-1.0)	0.007	1.0 (0.8-1.2)	0.904
PLT[b]	1.0 (0.9-1.1)	0.174	1.0 (0.9-1.1)	0.218	0.9 (0.8-1.0)	0.139

BM blast[d]	1.2 (1.0-1.3)	0.007	1.2 (1.1-1.4)	0.001	1.1 (0.9-1.3)	0.362
Nonintensive induction	2.7 (1.7-4.4)	<0.001	3.2 (1.9-5.5)	<0.001	1.3 (0.3-5.7)	0.714
Cytogenetic abnormality	0.9 (0.6-1.5)	0.789	0.9 (0.5-1.4)	0.550	0.6 (0.2-1.6)	0.311
MRD positivity after first consolidation			2.5 (1.5-4.3)	0.001	4.9 (2.2-11.2)	<0.001
Number of gene mutations	1.0 (0.9-1.1)	0.424	1.1 (1.0-1.2)	0.104	1.0 (0.9-1.2)	0.590
MRG mutations	1.0 (0.5-1.5)	0.392	0.5 (0.2-1.4)	0.177	0.3 (0.04-2.4)	0.266
MRC	1.0 (0.5-2.0)	1.000	1.1 (0.5-2.5)	0.753	1.6 (0.5-5.2)	0.468
WT1 mutation	1.3 (0.8-2.0)	0.228	1.3 (0.8-2.1)	0.304	1.1 (0.5-2.6)	0.826
GATA2 mutation	0.9 (0.6-1.5)	0.808	1.1 (0.7-1.8)	0.733	1.3 (0.6-2.9)	0.567
NRAS mutation	1.1 (0.6-1.9)	0.710	0.8 (0.4-1.6)	0.524	0.7 (0.2-2.3)	0.544
TET2 mutation	1.0 (0.6-1.8)	0.941	1.3 (0.7-2.4)	0.469	2.1 (0.8-5.5)	0.144
KIT mutation	1.3 (0.6-3.0)	0.534	1.8 (0.8-4.2)	0.160	3.1 (0.9-10.5)	0.163
FLT3-ITD mutation	1.3 (0.5-3.1)	0.622	1.9 (0.8-4.8)	0.059	1.5 (0.2-11.3)	0.694
CSF3R mutation	1.2 (0.5-2.8)	0.612	1.5 (0.6-3.6)	0.410	1.8 (0.2-14.0)	0.564
IKZF1 deletion/mutation	2.4 (1.3-4.5)	0.006	3.4 (1.8-6.5)	<0.001	1.5 (0.5-4.4)	0.432
DNMT3A mutation	1.4 (0.6-3.2)	0.405	2.1 (0.9-4.8)	0.082	1.8 (0.4-7.8)	0.407

BM, bone marrow; CI, confidence interval; HGB, hemoglobin; HR, hazard ratio; MRC, myelodysplasia-related cytogenetic abnormalities; MRD, measurable residual disease; MRG, myelodysplasia-related gene mutations; PLT, platelets; Ref. reference; WBC, white blood cell.

[a] Linear with estimates of HRs for every increase of 10 years.

[b] Linear with estimates of HRs for every decrease of 10 × 10^9/L.

[c] Linear with estimates of HRs for every increase of 10 g/L.

[d] Linear with estimates of HRs for every increase of 10%.

Supplemental Table S3. Multivariate analyses of outcomes

	**EFS**		**RFS**		**Survival**	
	HR (95%CI)	*P* value	HR (95%CI)	*P* value	HR (95%CI)	*P* value
WBC ×10^9/L[a]	1.9 (1.3-2.9)	0.002	2.5 (1.4-4.5)	0.003	2.6 (1.1-5.8)	0.024
HGB ≤ 72 g/L			2.9 (1.6-5.2)	<0.001		
Nonintensive induction	3.3 (2.0-5.4)	<0.001	3.9 (2.2-6.9)	<0.001		
MRD positivity after first consolidation			2.7 (1.4-4.3)	0.002	5.2 (2.3-11.9)	<0.001
FLT3-ITD mutations			3.1 (1.2-7.9)	0.018		
IKZF1 mutations and deletions	1.9 (1.3-2.9)	0.002	3.2 (1.6-6.5)	0.001		

[a]WBC count, EFS: ≥ 32 ×10^9/L; RFS: ≥ 69×10^9/L; OS: WBC ≥ 45 ×10^9/L.

CI, confidence interval; HR, hazard ratio; WBC, white blood cell; HGB, Hemoglobin; MRD, measurable residual disease.

Supplemental Table S4. Multivariate analyses of outcomes in patients receiving intensive induction therapy

	EFS		RFS		Survival	
	HR (95%CI)	*P* value	HR (95%CI)	*P* value	HR (95%CI)	*P* value
WBC, $\times 10^9$/L[a]	1.1 (1.0-1.2)	<0.001	1.1 (1.0-1.2)	<0.001		
MRD positivity after first consolidation			3.0 (1.6-5.6)	0.001	6.0 (2.6-13.8)	<0.001
FLT3-ITD mutations			2.9 (1.0-8.2)	0.049		
IKZF1 mutations and deletions	2.8 (1.5-5.4)	0.002	4.5 (2.3-9.1)	<0.001		

[a] Linear with estimates of HRs for every decrease of 10 × 10^9/L.

CI, confidence interval; HR, hazard ratio; WBC, white blood cell; MRD, measurable residual disease.

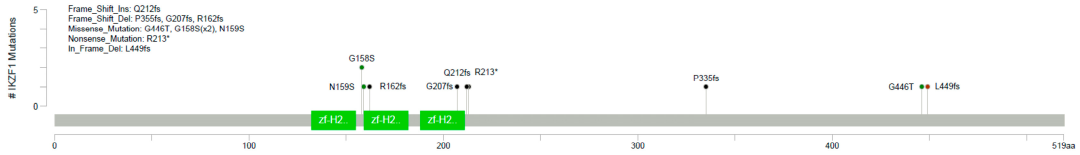

Supplemental Figure S1. Lollipop plot illustrating *IKZF1* mutations

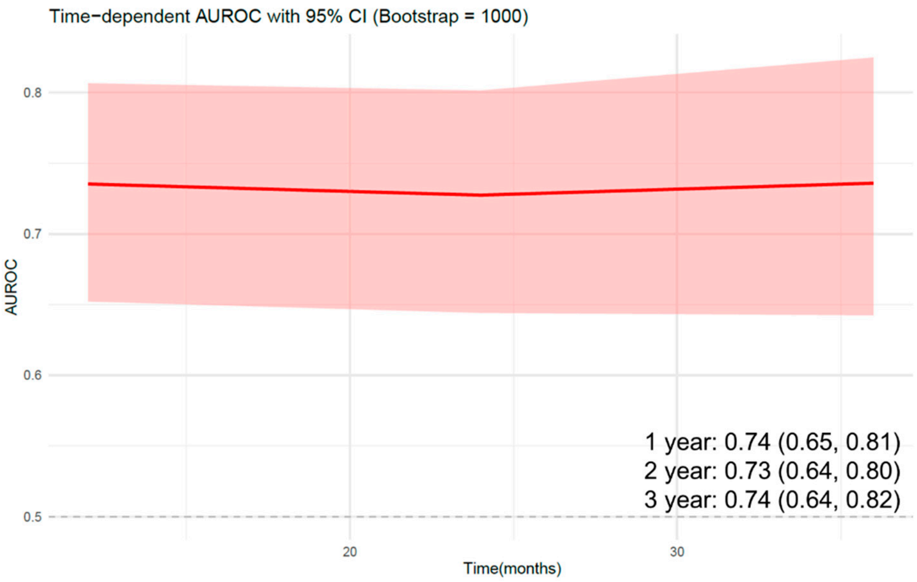

Supplemental Figure S2. ROC curves of the risk stratification for the 1-, 2- and 3-year

probabilities of RFS
